# Geo-spatial analysis of individual-level needle and syringe coverage in Melbourne, Australia

**DOI:** 10.1371/journal.pone.0209280

**Published:** 2018-12-14

**Authors:** Daniel O’Keefe, Anna Wilkinson, Campbell Aitken, Paul Dietze

**Affiliations:** 1 Behaviours and Health Risks, Burnet Institute, Melbourne, Victoria, Australia; 2 School of Public Health and Preventive Medicine, Monash University, Melbourne, Victoria, Australia; University of New Mexico Health Sciences Center, UNITED STATES

## Abstract

Distance to health services is known to be negatively associated with usage and needle and syringe programs (NSPs) for people who inject drugs (PWID) are no different. Australia has a mixture of NSP modalities (primary or secondary fixed-site NSPs), which may present unique barriers to access. In this study, we explore 1) the effect of distance to NSPs on individual-level needle and syringe coverage, and 2) differences in coverage dependent on NSP modality. Using data from 219 PWID in an ongoing cohort study in Melbourne, Australia, we measured the straight-line distance from participants’ residence to their nearest primary or secondary fixed-site NSP. We analysed the relationship between geographical distance and coverage via regression analysis. The median distance to any type of NSP was 1872 metres. Regardless of service type, 52% of participants lived within 2 kms of a fixed-site NSP and 87% lived within 5 kms. We found no association between distance to NSPs and syringe coverage or a significant difference in coverage by nearest service type. Our findings suggest that the number and distribution of NSPs in Melbourne, Australia caters adequately for the population of PWID.

## Introduction

Needle and syringe programs (NSPs), which provide sterile needles and syringes (hereafter “syringe/s”) to people who inject drugs (PWID), are an effective public health intervention to support the reduction of injecting risk behaviours (such as receptive sharing of unsterile syringes) and blood-borne virus (BBV) transmission, particularly HIV transmission [1–3], but geographical areas without NSPs have been shown to have higher rates of syringe sharing and BBV prevalence than areas with NSPs [4, 5].

Australian NSPs have a mix of modalities: primary NSPs (fixed-site, free injecting equipment, and PWID-centred access through trained personnel and adjunct services such as opioid substitution therapy (OST) prescribers onsite) or secondary NSPs (syringe distribution through health services such as hospitals or community health centres). Both primary and secondary NSPs can supplement their fixed-site activities with mobile syringe delivery and syringe vending machines (SVMs) for out-of-hours syringe dispensation. The third modality is privately-owned retail pharmacies that sell injecting equipment. Together, these three modalities mean Australia has over 3000 syringe distribution outlets. In 2016, there were, 102 primary fixed-site NSPs and 786 secondary fixed-site NSPs [6].

Health services are most used by local clients [7, 8] and this is true for NSPs [9–13]. Rockwell et al. (1999) tested the relationship between walking distance to NSPs, service use and injecting risk in New York City [11]. PWID living within a 10-minute walk of an NSP had increased odds of “typically” obtaining sterile syringes from NSPs and decreased odds of receptive syringe sharing [11]. However, this study was conducted at a time in New York when NSPs were few in number and possession of syringes without registration with an NSP was illegal [11]. Even so, this person-level finding has been supported by additional research exploring geographical effects of NSP utilisation [12, 13]. For example, higher density of services has been associated with a reduction in injecting risk [10]. However, even areas with a high number of services may experience barriers, such as police presence near NSPs [9, 14]. Furthermore, the different service types making up Australia’s mixture of NSP modalities present their own unique barriers to access. For example, the cost of syringes at pharmacies represents an immediate barrier to acquisition [15] for impoverished PWID, and some PWID prefer not to use NSPs due to their overt association with injecting drug use [12]. Whilst primary and secondary fixed-site NSPs perform essentially the same sterile syringe dispensation function, they have important differences and therefore potential barriers. Secondary fixed-site NSPs are non-specialised services generally located within community health centres or hospitals, which can represent an anonymity concern for PWID, particularly in rural areas with smaller populations [16]. Even so, Australian PWID overwhelmingly report acquiring syringes from primary/secondary fixed-site NSPs. In a national surveillance survey of PWID, 94% reported acquiring syringes (non-exclusively) from NSPs; the next most common source (17%) was pharmacies [17]. It is worth noting that in contrast to many US contexts, Australian primary/secondary NSP policy allows for unlimited syringe distribution, meaning Australian PWID can acquire many more syringes, therefore requiring less NSP visits to cover their injecting episodes and allowing them plan their injections more effectively [18]. Also, the possession of unused needles/syringes in Australia (excluding Western Australia) is legal [19], however, it has previously been reported that police presence near NSPs creates a significant disincentive to NSP access for PWID [12].

Despite these differences between primary and secondary NSPs, there is little research comparing the acceptability and effectiveness between these modalities. Fisher et al. (2017) conducted cross-sectional analysis of rural PWID in central New South Wales, Australia [20]. In these locations, study participants had no access to primary fixed-site NSPs. Despite higher injecting frequency than reported in national surveys, Fisher et al.’s rural PWID reported comparable levels of syringe acquisition and lower past month receptive syringe sharing [17, 20, 21]. Furthermore, approximately 90% reported a preference for syringe acquisition from NSPs, compared to pharmacies or SVMs [20]. In contrast, Day et al. reported rural PWID obtained their sterile syringes from a much wider variety of sources than their metropolitan counterparts [22]. If PWID are reluctant to collect sterile syringes from particular sources, this may reduce their syringe coverage, thereby increasing their risk of receptive syringe sharing and BBV transmission. Coverage is an important measure of NSP effectiveness [23], and the non-use or inconsistent use of NSPs has been associated with insufficient coverage at the individual-level [15, 24]; therefore, greater distance between individuals and NSP outlets, may lead to reduced coverage.

In this study we explore the straight-line distance from PWIDs’ residences to their nearest primary or secondary fixed-site NSP, and analyse the relationship between this distance and individual-level syringe coverage in Melbourne, Australia. Specifically, we aim to:

1. describe and analyse the association between geographical distance to a sterile syringe source and individual-level syringe coverage;
2. examine the differences in individual-level syringe coverage between participants whose nearest service type is a primary or secondary fixed-site NSP.

## Methods

### Data sources

We drew data from three separate data sources:

1. the Melbourne injecting drug user cohort (MIX) study dataset; longitudinal interview data for 757 participants, recruited as “regular” PWID. Data from recruitment in 2008 until December 2016 was available for this study;
2. the MIX cohort participant contact database; a Microsoft Access tool, used to store participant contact information (telephone numbers, addresses, etc.) for the purpose of contact tracing;
3. publicly available data for each needle and syringe dispensing outlet in Victoria, Australia (N = 519), provided by the Australian State Government Department of Health and Ageing [25], accessed in October 2015. The data at the time categorised outlets as “primary fixed-site” (n = 11 sites), “secondary fixed-site” (n = 176 sites) or “pharmacy” (n = 331 sites). One primary enhanced health service targeting PWID in central Melbourne operates syringe delivery outreach by foot patrol; in analysis, this service was considered to be a primary fixed-site NSP. The dataset includes the address of each outlet and the latitude and longitude of this address.

The MIX study has been described in detail elsewhere [26]. Briefly, participants are administered an annual structured questionnaire. Recruitment of the original 688 MIX participants occurred between 2008 and 2010; an additional 69 participants were included in the cohort in 2011 via past involvement in the Networks II cohort [27], resulting in 757 participants. Both MIX and Networks II sought to recruit PWID who injected regularly. The characteristics of the cohorts at baseline (2005 for Networks II) were comparable [28]. Eligibility criteria for the original MIX cohort were being aged 18–30 years and reporting injecting heroin and/or methamphetamine regularly (at least once a month in the six months prior to recruitment [24]. All participants completed written informed consent. Ethics approval was received by the Victorian Department of Health and Human Services and Monash University Human Research Ethics Committees.

Using the MIX contact database, we identified the most recent residential addresses (up until December 2016) for all 757 participants. We manually verified address information and excluded participants whose addresses were specified as postal (referring to an address a participant directs mail to, rather than an address they live at) rather than residential (n = 23), those who stated they had no fixed address or gave invalid address data (n = 28), and those specifying their mailing address as a post office box or a community service (such as an NSP) (n = 15). Interstate or international addresses were also excluded from analysis (n = 20). The remaining 671 participants with valid address data were matched with their most recent interview data within the MIX dataset. Visual inspection revealed a further 50 participants with incorrectly matched interview and address data, which we excluded from analysis. In total, 621 participants had both valid address information and correctly matched interview data up until December 2016.

### Geo-coding distance from participant address to syringe distribution services

**Fig 1:**
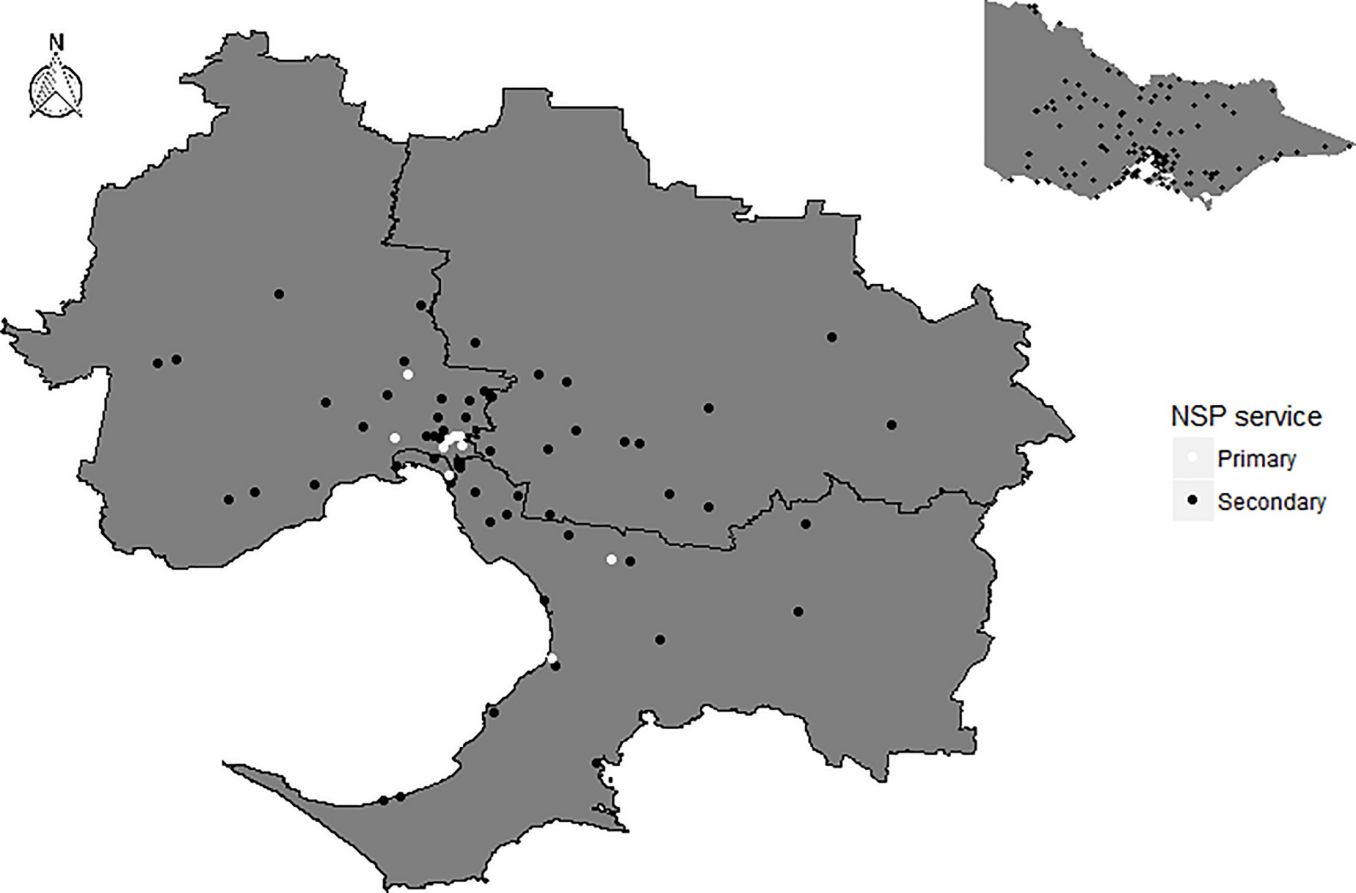
Geographic distribution of primary/secondary fixed-site NSPs in Melbourne, Australia*.

The latitude and longitude of both participant address and service address were mapped using mapping software–ArcGIS Online and ArcMap V.10.4 (Esri, CA, USA). Our altered map of Victoria in Fig 1 was drawn from the Victorian government’s Vicmap data admin website—creative commons license [29]. Services were stratified into “primary fixed-site NSP” and “secondary fixed-site NSP” (according to Victorian State Government Department of Health classification). Straight-line distance (“as the crow flies”) was measured from each participant’s address to: 1) their nearest primary fixed-site NSP, 2) their nearest primary fixed-site NSP *OR* their nearest secondary fixed-site NSP (near table function in ArcMap). This process created two distance measurements per participant. Participants were then categorised as living closest to either a primary fixed-site NSP or a secondary fixed-site NSP. All distance was measured in metres.

Due to the abundance of syringe selling pharmacies in Melbourne, this meant that the closest source of syringe acquisition for nearly all participants was a pharmacy. Further, pharmacies are fundamentally different to primary/secondary NSPs for two reasons: 1) they do not offer the same health services as NSPs, including those listed above, such as vaccinations and blood tests. Further, primary NSPs are specifically designed to cater to the social/health needs of PWID, such as offering drug counselling, OST prescription and housing support–services that pharmacies are simply unable to offer; 2) Pharmacies sell needles/syringes; this deters many PWID, especially because they can acquire unlimited syringes for free from the numerous NSPs throughout Melbourne. Consequently, we felt the inclusion of pharmacies would bias our measurement of distance, particularly considering that recent national surveillance of Australian PWID showed that only 12% of Victorian PWID (lower than the reported national prevalence of 17%) reported using a pharmacy as a source of syringe acquisition in the six months prior to survey [30].

### Individual-level needle and syringe coverage

The MIX questionnaire includes questions to record the four primary coverage parameters (syringe acquisition, peer-to-peer syringe distribution, sterile syringe stockpiling and injecting frequency):

- *In the last* ***two weeks*** *how many new (needles and) syringes in total did you get*?
- *In the last* ***two weeks*** *how many new (needles and) syringes did you give away or sell to others*?
- *At the moment*, *how many (needles and) syringes do you have stored away (at home or in the car etc*.*)*?

Past week injecting frequencies for 18 different drug types were summed to create a total injecting frequency variable. Responses to each question were recorded as continuous variables. Past week injecting frequency was doubled to match the timeframe specified in the other coverage questions, creating an estimate of past two-week injecting frequency.

We adapted the method of calculating individual-level syringe coverage devised by Bluthenthal et al. [31] and refined by McCormack et al. [18]. The number of syringes distributed was subtracted from the number of syringes acquired, and the number of stockpiled syringes was added; the result was divided by the past-two-week estimate of injecting frequency and then multiplied by 100, giving a percentage of injecting episodes “covered” by a sterile syringe. The coverage formula is presented below.

$$\frac{\left(syringes\;acquired-syringes\;distributed+syringes\;stockpiled\right)}{\left(past\;week\;injecting\;frequency\;x\;2\right)}\;x\;100$$

Coverage was calculated only for those participants with interview data occurring after February 2015 (when the necessary coverage questions were included within the MIX questionnaire; excluding 158 participants from analysis), those with valid data for each coverage parameter and those who reported injecting within the two weeks prior to interview. Ultimately, 219 participants had valid data for coverage calculation; 33% of all coverage observations were missing (92% due to injecting abstinence). Data for these 219 participants were used in final analysis.

Coverage was calculated as a continuous measure, though also treated as a dichotomous outcome; either sufficient (≥100% of injecting episodes covered by a sterile syringe) or insufficient (<100%) coverage [15, 32].

### Statistical analysis

To test the association between distance and individual-level coverage as a continuous variable, we constructed a multivariable linear regression model, using continuous coverage as the outcome, and the continuous distance to nearest primary *OR* secondary fixed-site NSP as the exposure sub-group of key interest. A separate multivariable logistic regression model tested associations with insufficient coverage, comparing those living closest to a primary fixed-site NSP vs. a secondary fixed-site NSP. Both models controlled for participants’ sex (female/male), employment status (not employed / employed [full time, part time/casual, student, home duties, other]), current accommodation status (unstable [boarding house, institutional accommodation, squat, homeless] / stable [owner occupied property, private rental property, public housing, boarding with family/friends]) and residence being inner metropolitan or outer metropolitan, as defined by governmental health catchment areas [33]. Due to the relatively small number of participants experiencing insufficient coverage, and this being the outcome of interest in our logistic regression model, we were limited in the number of potential covariates for inclusion. We therefore chose those covariates we felt might most affect the relationship between distance to NSPs and coverage *a priori*. We similarly restricted the number of covariates in the linear regression model to make the models comparable. Our models were considered fixed after selection of covariates, with no stepwise elimination being performed. The Hosmer-Lemeshow test was used to determine goodness-of-fit for the logistic regression model. Summary statistics are reported for all regression analyses. Significance was set at p<0.05. Statistical analysis was conducted in Stata13 (Statcorp LP, TX, USA). Graphics were created in RStudio 1.0.143 (RStudio, Inc., MA, USA).

## Results

### Sample demographics

Our amended sample of 219 participants was mainly male (61%), unemployed (79%) and living in stable accommodation (82%). Eighty-five per cent of participants reported injecting heroin in the month prior to interview, whilst 48% reported injecting methamphetamine (these percentages were not mutually exclusive). Heroin was the drug most commonly injected in the month prior to interview (72%), followed by methamphetamine (21%). Fifty-four per cent of participants reported currently being on some form of OST (mostly methadone). Participants had an average age of 35 years at time of interview.

As shown in Table 1, our amended sample of 219 participants for analysis was mostly representative of the reduced sample (n = 621), although the reduced sample had less participants on OST (47% vs. 54%). The demographic and drug use statistics of both samples largely accorded with those of the original cohort at baseline, as reported by Horyniak et al. (2013) [26], although noticeably more participants reported injecting heroin at baseline compared to our contemporary sample. This may be a result of the changing drug market in Melbourne, which has recently seen dramatic increases in methamphetamine purity [34].

**Table 1:** Demographic and drug use differences between MIX cohort samples in Melbourne, Australia (2008–2016).

Characteristic	Baseline cohort (Horyniak et al.)	Reduced sample (n = 621)	Final amened sample (n = 219)
*Sex (male)*	67%	65%	61%
*Employment status (unemployed)*	86%	81%	79%
*Accommodation status (stable)*	81%	83%	82%
*Currently on OST*	35%	47%	54%
*Age (median)*	28 ^$^	32	35
*Injected heroin (past month)*	82%	83%*	85%
*Injected methamphetamine (past month)*	27%	44%*	48%
*Abstained from injecting (past month)*	25%	19%	-

* Amongst those injecting (n = 498)

^$^ Age at baseline (2008–9)

### Distance by nearest service type

Median distances to NSPs, stratified by service type, are shown in Table 2. The median distance to any type of fixed-site NSP was 1872 metres (IQR: 1131, 3370). Fifty-two per cent of participants lived within 2km of any service type. Eighty-seven per cent lived within 5km of any service type. Those living closest to a primary fixed-site NSP lived a median 1859 metres (IQR: 673, 3464) from the service, whilst those living closest to a secondary fixed-site NSP lived a median 1914 metres (IQR: 1236, 3203) away. For those living closest to a secondary fixed-site NSP, the median *additional* distance to a primary fixed-site NSP was substantial, at 4405 metres (IQR: 1987, 11202).

We compared descriptive distances between our reduced sample (n = 621) and our final sample for analysis (n = 219) and across all measures found the statistics to be nearly identical.

**Table 2:** Distance between participant residence and nearest NSP service type in Melbourne, Australia (2015–2016), stratified by participant’s nearest service type.

	All participants (n = 219)	Those closest to a primary fixed-site NSP (n = 50)	Those closest to a secondary fixed-site NSP (n = 169)
**Median (IQR) distance (metres)**			
*Primary*	5754 (3181, 12505)	1859 (673, 3464)	7944 (3709, 15241)
*Primary/Secondary*	1872 (1131, 3370)	-	1914 (1236, 3203)
**Distance to primary, n (%)**			
*≤1 km*	17 (8%)	15 (30%)	2 (1%)
*1–2 kms*	19 (9%)	12 (24%)	7 (4%)
*2–5 kms*	66 (30%)	18 (36%)	48 (29%)
*≥5 kms*	117 (53%)	5 (10%)	112 (66%)
**Distance to primary/secondary, n (%)**			
*≤1 km*	45 (20%)	-	30 (18%)
*1–2 kms*	70 (32%)	-	58 (34%)
*2–5 kms*	76 (35%)	-	58 (34%)
*≥5 kms*	28 (13%)	-	23 (14%)

NSP: Needle and syringe program; IQR: Interquartile range

Primary NSP refers to services specifically tailored to PWID, with health/social services additional to needle/syringe distribution. Secondary NSP refers to needle/syringe distribution attached to other non-specific health services, such as community health centres/hospitals, etc.

### Relationship between geographical distance and coverage

Median continuous coverage was 250% (IQR: 100%–650%), meaning that across the sample, participants generally acquired 2.5 sterile syringes per injecting episode. Continuous coverage was similar for participants living closest to a primary fixed-site NSP (median: 204%) and those living closest to a secondary fixed-site NSP (median: 250%).

In bivariable testing, for every additional metre to any service type, coverage increased by 0.01 percentage points; this association was not significant (p = 0.58). The effect size or significance of the association did not change in the multivariable model, after accounting for other (also non-significant) covariates (Table 3).

**Table 3:** Linear regression testing association between distance to nearest NSP service and continuous coverage amongst PWID in Melbourne, Australia (2015–2016).

	Coefficient (95% CIs)	p-value
**Distance to primary or secondary fixed-site NSP**		
	0.01 (-.05, 0.07)	0.762
**Sex**		
*Female*	1	
*Male*	39.59 (-247.01, 326.20)	0.786
**Employment status**		
*Unemployed*	1	
*Employed*	198.54 (-150.10, 547.18)	0.263
**Accommodation status**		
*Unstable*	1	
*Stable*	-141.30 (-223.55, 506.15)	0.446
**Inner vs. outer metro**		
*Inner metro*	1	
*Outer metro*	-56.27 (-835.06, 722.52)	0.887

NSP: Needle and syringe program, 95% CIs: 95% Confidence intervals

Number of observations: 211; Prob(chi^2^): 0.841; R^2^: 0.01

### Dichotomised coverage: Those living closest to primary vs. secondary fixed-site NSP

After dichotomising coverage, 169 participants (77%) were sufficiently covered and 50 (23%) were insufficiently covered. The results from the multivariable logistic regression model, testing the associations with insufficient coverage (outcome), were all non-significant (Table 4). Specifically, we found no significant relationship between coverage and those living closest to a primary vs. a secondary fixed-site NSP (AOR: 1.09, 95% CI: 0.51, 2.36, p-value: 0.818).

**Table 4:** Logistic regression associations between independent variables and insufficient coverage amongst PWID in Melbourne, Australia (2015–2016).

	Total (n = 219)	Insufficient coverage (n = 50)	AOR (95% CIs) with insufficient coverage	AOR p-value
**Sex**				
*Female*	85 (39%)	17 (34%)	1	
*Male*	134 (62%)	33 (66%)	1.34 (0.68, 2.64)	0.395
**Employment status**				
*Unemployed*	172 (79%)	38 (76%)	1	
*Employed*	47 (21%)	12 (24%)	1.30 (0.60, 2.84)	0.507
**Accommodation type**				
*Stable*	178 (81%)	39 (78%)	1	
*Unstable*	41 (19%)	11 (22%)	1.38 (0.62, 3.07)	0.434
**Inner metro vs. outer metro**				
*Inner metro*	203 (96%)	48 (96%)	1	
*Outer metro*	8 (4%)	2 (4%)	1.07 (0.20, 5.85)	0.934
**Primary vs. secondary fixed-site NSP**				
*Primary*	50 (23%)	11 (22%)	1	
*Secondary*	169 (77%)	39 (78%)	1.09 (0.51, 2.36)	0.818

NSP: Needle and syringe program, AOR: Adjusted odds ratio, 95% CIs: 95% Confidence intervals

Number of observations: 211; Prob(chi^2^): 0.869; Pseudo R^2^: 0.008

## Discussion

In our sample, 52% of participants lived within 2 km of a primary or secondary fixed-site NSP and 87% lived within 5 km. The median distances to a primary fixed-site NSP or a secondary fixed-site NSP, for those living closest to either of these services, were almost identical (1.8 km vs. 1.9 km respectively).

We explored the effect distance to NSPs had on individual-level syringe coverage in multiple ways and found no significant effect. Individual-level syringe coverage did not differ for participants whose closest source of sterile syringes was a primary fixed-site NSP or a secondary fixed-site NSP.

Distance to services is known to be negatively associated with usage. Our findings suggest that the number and spatial distribution of services in Melbourne, Australia facilitate easy sterile syringe access. The number of services providing sterile syringes, therefore, potentially caters adequately for the population of PWID. Cooper et al. have published extensively on the geographic influences on syringe access and injecting risk [9, 10, 35], showing greater density of services increases the odds of injecting with a sterile syringe [10]. High service density not only minimises travel time to services, but provides multiple options for syringe acquisition. In our analysis, the median distance to services was small, access also being especially improved due to the widespread and efficient public transport links in inner metropolitan Melbourne, where the majority of our sample resides. Moreover, Melbourne’s NSPs operate with an unrestricted syringe dispensation policy, allowing PWID to acquire as many sterile syringes as they like in a single visit without the need to return unsterile syringes. This contrasts with more restrictive dispensation policies in locations internationally which have been shown to have deleterious effects on coverage [36]. The impact of distance is presumably lessened if PWID are able to acquire as many syringes as needed, thereby reducing the number of times they need to visit an NSP in order to cover their injecting episodes.

The distinction between primary and secondary fixed-site NSPs is important. Primary fixed-site NSPs offer a range of adjunct, drug-related services (both social and health-related), in supportive environments populated by specialised staff. Few secondary fixed-site NSPs (especially those co-located at hospitals) provide similarly tailored services. It is therefore pleasing to see that the nature of the nearest NSP–primary or secondary–had no impact on syringe coverage levels. This suggests that though the services primary and secondary fixed-site NSPs offer may differ, the common factor of free, unlimited syringe dispensation means users’ coverage is not appreciably different. Even so, barriers to accessing NSPs do exist, as demonstrated within our interview data: regardless of closest service type, 17% of participants reported not using NSPs as their *usual* source of sterile syringes, reporting alternate syringe sources, such as pharmacies, friends or dealers. Whilst the non-use or inconsistent use of NSPs has previously been associated with insufficient coverage [15, 37], it is true that NSPs are simply not acceptable to all PWID [12, 38].

How to enable sufficient acquisition of sterile syringes may not then be a question of service density in Melbourne, but rather the accessibility and acceptability of services more broadly. Structural barriers to NSP access beyond geographic distance have been noted, such as opening hours [12, 39], experiencing stigma from NSP staff [12] and, as previously noted, police activity, which may counteract the positive effects of high spatial access [9]. Many NSPs supplement their fixed-site service with SVMs, which are effective at increasing temporal and geographic access to sterile injecting equipment [40]. Approximately 300 SVMs currently operate in Australia [6], yet only six are located in Victoria [25], the first being introduced in 2014. At present, half of these SVMs are concentrated in a single area of outer Melbourne, but the effect of distance to SVMs could be analysed using methods similar to those employed in this paper in the future when there are a greater number of more dispersed SVMs. Finally, many clients report experiencing stigma from NSP staff that creates an immediate barrier to access [12]. The overtness of stigma may differ across service types [41, 42] if staff are not specially trained in drug and alcohol issues or encounter PWID infrequently. Stigma reduction training has demonstrated positive results [43], and is recommended for all NSP staff. If the concentration of syringe distribution services in Melbourne is sufficient to eliminate the effect of distance on coverage, then minimising other barriers to access is important.

### Limitations

We measured distance in a straight line from participants’ residence to services, thereby underestimating true travel distance to acquire sterile syringes. However, it is likely that the degree of underestimation was evenly distributed across participants and locations. We felt straight line distance would account for the various methods PWID may reach an NSP: they may walk (often taking laneways or back streets), they may take trains or use the tram or bus system (Melbourne has an extensive and efficient public transport system) or they may drive (though most of our participants do not drive). All of these methods would require a different path to be taken to the nearest NSP, therefore, straight line distance may average out these various methods of reaching a particular destination. Also, we made assumptions about service utilisation. Living closest to a particular service does not necessarily translate into use of that service, especially if another equivalent service is nearby.

We recognised that the presence of the primary outreach NSP in Melbourne’s CBD may have biased our results, as NSP staff respond to call-outs from PWID in the area. However, of the 50 participants whose closest NSP was a primary NSP, none had this particular service as their nearest NSP.

The dataset of service locations was accessed in October 2015. Our analysis assumed these locations existed consistently for the timespan of our interview dataset (2009–16). This assumption could not be validated, although we believe any potential impact of change of NSP location would be small. First, we know that the addresses of the primary NSPs have remained constant. Second, bias would occur only via change of location for a participants nearest secondary NSP, meaning any change of location (if any change did indeed occur) would affect only a small number of participants.

Our sample contained a very small proportion of outer-metro participants (approximately 4%), limiting generalisability beyond inner-metro PWID and certainly preventing extension of our findings to rural or regional PWID. Further, the nature of recruitment for the MIX study, occurring primarily in inner-Melbourne suburbs, meant our sample had greater NSP access. However, this does not, by default, mean that our participants lived close to the NSPs they were recruited from, particularly because MIX was recruited through the small number of primary NSPs and not the far greater in number of secondary NSPs.

The level of NSP service provision in Australia is high and dispensation policies are liberal compared to many other countries, where it is expected that distance would have a powerful negative effect on coverage. Consequently, the applicability of our results to locations with fewer services and more restrictive dispensation policies is doubtful.

### Conclusions

We explored the impact of geographical distance to primary and secondary fixed-site NSPs on individual-level syringe coverage. We found no association between distance and coverage, either as a continuous measure or when comparing different service types. We hypothesise that in our sample, distance had no substantial effect on individual-level coverage due to the density of sterile syringe distribution sources and unrestricted syringe dispensation policies in Melbourne. If this density means service distance is of little relevance to sterile syringe acquisition in Melbourne, improving other elements of access is recommended to further reduce injecting risk and consequent BBV transmission.
